# Emergency-Driven Multiple Simultaneous Invasive Procedures in Haemophilia

**DOI:** 10.3390/life14091172

**Published:** 2024-09-18

**Authors:** Cristina Emilia Ursu, Margit Șerban, Jenel Marian Pătrașcu, Daniel Coriu, Jenel Marian Pătrașcu, Ioana Ioniță, Adina Trăilă, Ciprian Tomuleasa, Delia Săvescu, Melen Brânză, Codruţ Ivan, Teodora Smaranda Arghirescu

**Affiliations:** 1 Onco-Hematology Research Unit, Romanian Academy of Medical Sciences, Children Emergency Hospital “Louis Turcanu” Timișoara, European Hemophilia Treatment Centre, 300011 Timișoara, Romania or emiliaursu@spitalcopiitm.ro (C.E.U.); or mserban2009@gmail.com (M.Ș.); 2 Department of Orthopedics, “Victor Babes” University of Medicine and Pharmacy Timișoara, 300041 Timișoara, Romania; jenelmarianp@yahoo.com; 3 2nd Clinic of Orthopedics and Traumatology, County Emergency Hospital ‘Pius Branzeu’, Nr. 2, 300041 Timișoara, Romania; 4 Hematology (Clinic and Laboratory) Discipline-Fundeni Clinical Institute, “Carol Davila” University of Medicine and Pharmacy, 020021 Bucharest, Romania; daniel_coriu@yahoo.com; 5 Department of Hematology and Bone Marrow Transplant, Fundeni Clinical Institute, 022328 Bucharest, Romania; melen.brinza@yahoo.com; 6 Department of Hematology, “Victor Babes” University of Medicine and Pharmacy Timișoara, 300041 Timișoara, Romania; ionita.ioana@umft.ro; 7 Medical Centre for Evaluation Therapy, Medical Education and Rehabilitation of Children and Young Adults, European Hemophilia Comprehensive Care Centre, 305100 Buziaș, Romania; adinatraila@yahoo.com; 8 Department of Hematology, Research Center for Functional Genomics and Translational Medicine, Iuliu Hațieganu University of Medicine and Pharmacy, 400012 Cluj Napoca, Romania; ciprian.tomuleasa@umfcluj.ro; 9 Laboratory Department, Children Emergency Hospital “Louis Turcanu” Timișoara, European Hemophilia Treatment Centre, 300011 Timișoara, Romania; delia.savescu@gmail.com; 10 Surgical Clinic I, Department of Surgery II, ”Victor Babeş” University of Medicine and Pharmacy Timişoara, Eftimie Murgu Square, No. 2, 300041 Timișoara, Romania; codrutivan@gmail.com; 11 Clinic of Surgery I, “Pius Brânzeu” Emergency County Clinical Hospital Timişoara, Liviu Rebreanu Blv. No. 156, 300723 Timișoara, Romania; 12 Department of Pediatrics, Division of Onco-Hematology, “Victor Babes” University of Medicine and Pharmacy Timișoara, 300041 Timișoara, Romania; sarghirescu@yahoo.com

**Keywords:** haemophilia, surgical emergency, simultaneous concomitant intervention

## Abstract

Despite the controversies regarding the appropriateness and justification of simultaneous bi- and multi-concomitant surgical procedures, this operative technique is increasingly undertaken for economic reasons. This paper discusses three cases of simultaneous interventions: two involving osteoarticular procedures and one involving a complex approach encompassing general and plastic surgery. The indications in emergency-driven cases are mandatory, life-saving, and limb-saving, and not subject to debate.

## 1. Introduction

The current state of medicine may be considered a real success story; it is witnessing a spectacular evolution worldwide, with a wide range of novel therapeutical products and approaches [1,2]. In the field of surgery, modern medical advances allow the expansion of a range of novel technological innovations and combined interventions. In the last decades, the number of simultaneous invasive procedures has increased. This chapter in interventional medicine (open surgery vs keyhole surgery, minor vs major surgery, elective vs emergency surgery, general, orthopaedic, cardiac, plastic, trauma, reconstructive surgery, etc.) is defined by simultaneous concurrent versus overlapping surgery [3,4]. Both are conducted on two elective patients in part at the same time, performed by the same primary attending doctor. The decision is driven by the advantages of allowing a more significant number of procedures in a given period by physicians in the same operating room, aiming to increase the throughput of clinic cases and the expert clinical staff. The other type of procedure, our current subject, is the bilateral or multiple (two or more) simultaneous concomitant interventions recorded by the same physician on the same patient under one single period of anaesthesia.

In the present years of the consolidated “golden era of haemophilia”, the tendency to perform elective programmed simultaneous surgery is also increasing in this provocative field. Spontaneous and traumatic joint bleeding are the hallmarks of haemophilia and are the most prominent clinical characteristics present in almost all cases. The statement that one joint bleed is too much is evidence-based; it can start the vicious circle for the development of chronic synovial and osteochondral deleterious damages [5]. Hemarthropathy is consequently the most significant cause of morbidity in persons with haemophilia (PwH). Nowadays, the revolutionary development of the therapeutical arsenal (recombinant factors of the first, second, third, and fourth generation, extended half-life products, non-factor replacement products) of haemophilia treatment has dramatically changed the evolution of the disease. Life expectancy and quality of life reached almost similar levels to non-hemophilic persons. Unfortunately, the great majority of PwH in the world (about 70%), with a medical history of lack of primary prophylaxis and life-long regular high-level personalized prophylaxis, are in a detrimental condition, with frequent pathological involvement of bilateral main joints [6]. As orthopaedic surgery is the only solution for those with hemartropathy who fail to respond to analgesics, orthotics, and physical therapy, the proportion of candidates for these types of interventions has constantly increased [7]. As the treatment of haemophilia is very expensive, the search has begun for cost-effective simultaneous or synchronous bilateral concomitant knee (TKA) or hip (THA) arthroplasty with the same anaesthesia performed by the same physician, a method already successfully performed in non-hemophilic patients; this method has now been initiated in PwH [8,9,10,11,12,13,14,15,16]. The main reason for the attempt of simultaneous bilateral interventions has been the cost-utility [17,18,19,20,21,22].

However, despite the many advantages of simultaneous programmed orthopaedic interventions compared to ranged procedures arthroplasty, this type of orthopaedic treatment remains controversial [10,11,12,15,17]. Doubtless, a significant reduction in expenditure is a reality, but we should not forget that patient safety must always take precedence over cost [7,23,24,25].

It is completely different from the situation in case of emergencies. Emergency-driven concomitant multiple invasive procedures are mandatory in these patients, and the prompt, timely surgical solution can have life- or limb-saving value. The literature review registered a high number of cross-sectional studies regarding this topic; in contrast to orthopaedic intervention, the number of reported emergencies driven simultaneously by non-orthopaedic ones is comparatively low [26,27,28,29].

We conducted a multi-institutional, unicentric, descriptive and analytical, retrospective study dedicated to surgery in haemophilia, based on a multi-institutional collaboration between various clinics of the “Victor Babeș” University of Medicine and Pharmacy from Timișoara (our European Hemophilia Treatment Center, orthopaedics, general surgery, neurosurgery, stomatology, etc.) as an example of a multi-institutional haemophilia comprehensive care centre.

## 2. Materials and Methods

We evaluated persons with haemophilia who underwent surgical intervention between June 2001 and June 2024, with a specific focus on those who had two or more simultaneous invasive procedures. Our goals were to:

- assess the type of haemophilia, comorbidities, age at the time of the intervention, type of emergency, type of intervention, and quantity of blood loss and transfusion;
- consider three main principles: costs (including treatment with coagulation factor products and hospital stay), early complications (such as infection, bleeding, and thrombotic events), and late complications (including infection, onset of inhibitors, and thrombosis), as well as outcomes (aseptic loosening and need for revision).

## 3. Results

In PwH, a large sample with a great diversity of 262 surgical interventions has been performed, and only three of them (1.15%) were simultaneous bi- and multi-concomitant procedures (Table 1). All three cases have been non-elective emergency driven: two needed an orthopaedic, and one needed a more complex abdominal and cutaneous intervention. Surgery was mandatory as limb-saving in the first two and life–saving in the third case (Table 1 and Table 2). We will present them in the following section.

**Figure 1:**
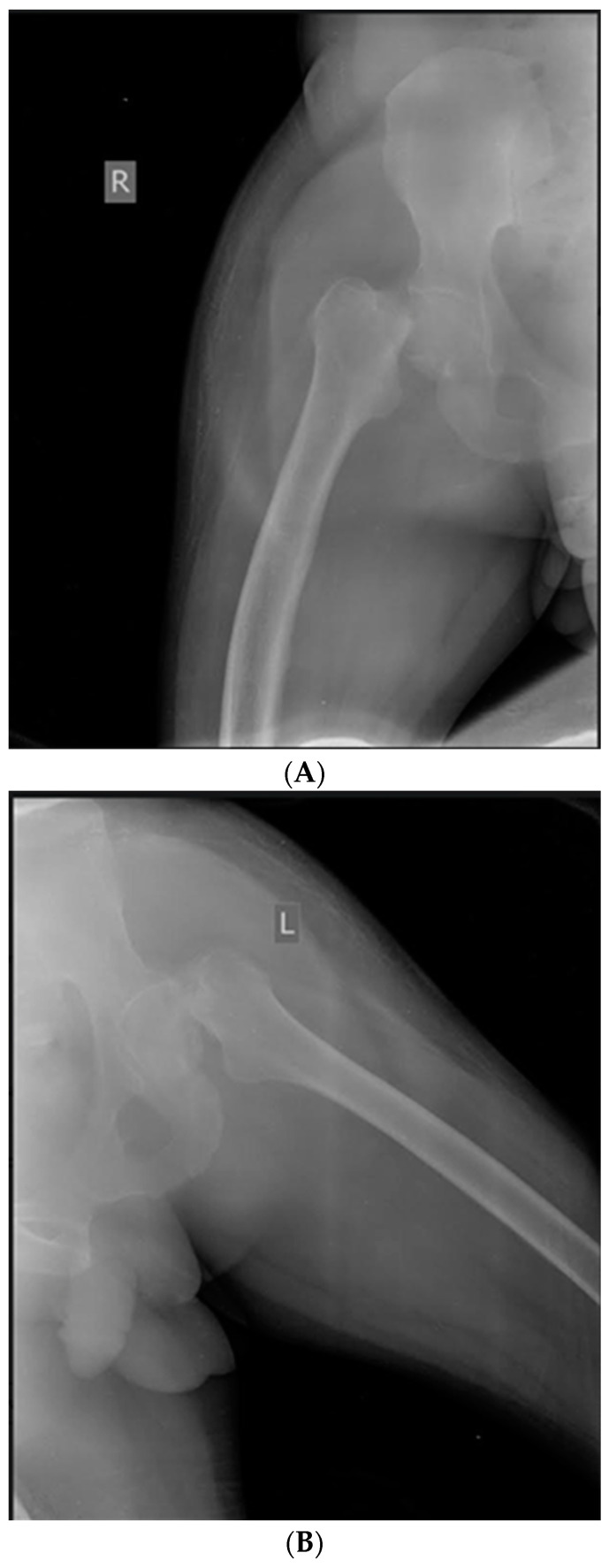

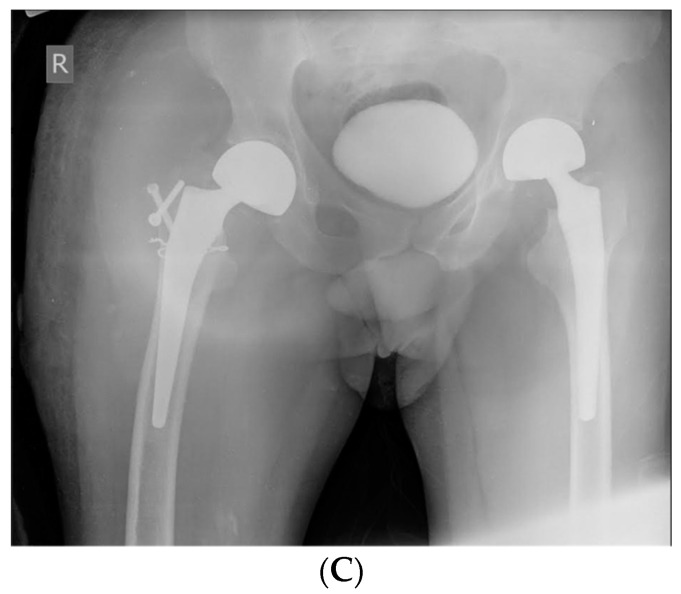
(**A**) Case 1—X-ray: Right femoral neck fracture—before surgery. (**B**) Case 1—X-ray: Left femoral neck fracture—before surgery. (**C**) Case 1—X-ray: Bilateral simultaneous hip arthroplasty, the right hip joint was complicated with pertrochanteric fracture and needed additional osteosynthesis with screws.

**Case 1.** Femoral neck fractures are common in the elderly population, who are frequently confronted with osteoporosis. Though rare, these fractures can also occur, mainly unilaterally, in younger individuals following a high-energy trauma. Only a few articles describe bilateral simultaneous femoral neck fractures as an injury resulting from seizures, and these were not assessed in PwH. We report a case of a bilateral femoral neck fracture in a 50-year-old male with severe haemophilia B (SHB) following a generalized convulsion. The patient was addressed to the emergency department for a generalized tonic-clonic seizure episode, and the patient was known and had been treated for epilepsy; he presented with evidence of SHB and multiple haemophilic arthropathies without a history of trauma or any comorbidities. On examination, the lower limbs were externally rotated; there were no distal neurovascular deficits, open wounds, or trauma evidence. Radiography revealed a bilateral femoral neck fracture (Figure 1A,B). Under rigorous hemostatic control, the orthopaedic surgeon team decided to intervene in one stage, and bilateral simultaneous hip arthroplasty was performed. The right hip joint was complicated with pertrochanteric fracture and needed additional osteosynthesis with screws (Figure 1C). Postoperatively, bedside hip range of motion exercises were started within days 2–3 after surgery, continuing with an enhanced rehabilitation program. The patient had a favourable outcome without any post-operative complications and was discharged after 30 days. Overall costs were EUR 86,856.05, and the factor concentrate accounted for 91.75% of expenditures (recombinant FIX, ~EUR 79,697.48). However, these two simultaneous major interventions had a decisive, favourable effect on the costs and rehabilitation of the patient. Due to a forceful contraction of muscles during an episode of generalised tonic-clonic seizures, a fracture or dislocation may develop.

**Figure 2:**
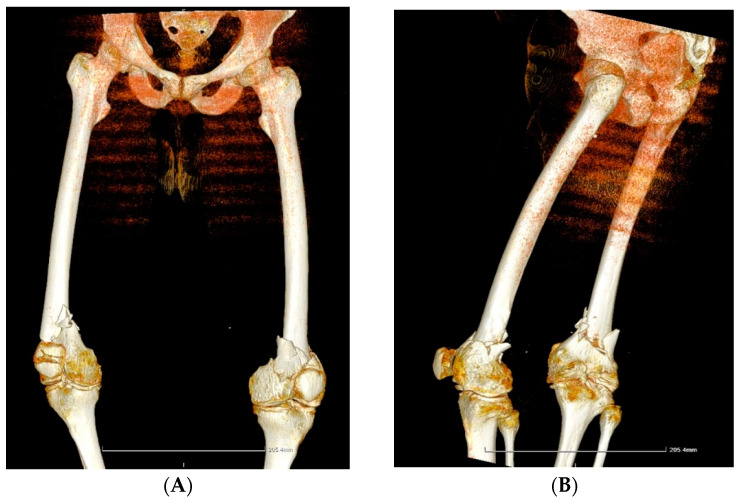

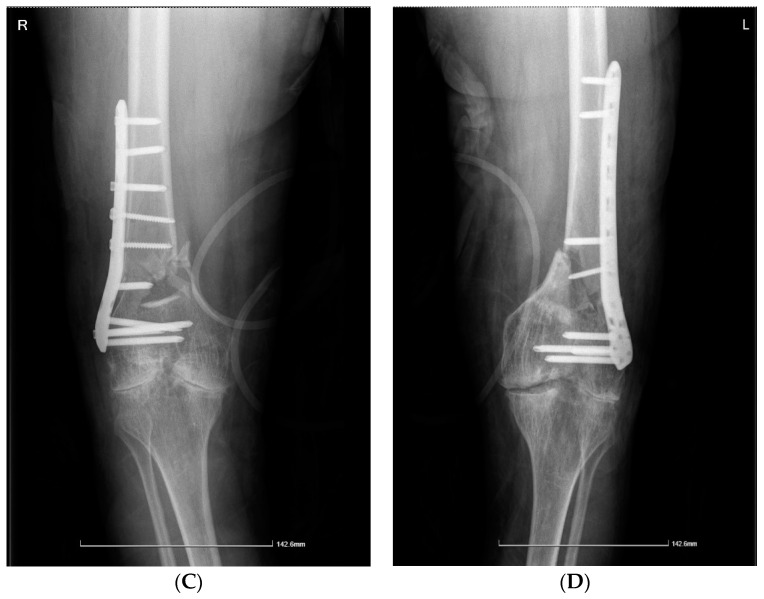
(**A**,**B**) Case 2—Computed tomography image: bilateral femoral supracondylar comminuted fracture. (**C**,**D**) Case 2—X-ray of right and left femur and knee. Open post-reduction status and internal fixation with present osteosynthesis material (plate with screws) at the level of the diaphysis and distal femoral epiphysis, following a femoral supracondylar comminuted fracture.

**Case 2.** A 39-year-old patient with severe haemophilia A, haemophilic arthropathies, and chronic viral hepatitis C, but no other comorbidities, was brought to the Emergency Unit after a car accident with acute pain and complete functional impotence of both legs. Upon examination, it was found that the patient had a bilateral femoral supracondylar fracture (Figure 2A,B). The patient underwent surgical intervention, specifically an open reduction and osteosynthesis with plates and screws on both legs (Figure 2C,D), while being treated with an extended half-life (EHL) FVIII concentrate to prevent and control bleeding. The total EHL FVIII required for the surgery and recovery was 63,500 IU. After three weeks of hospitalization, the patient’s condition improved, and he was discharged with the following instructions: 8–12 weeks of rest, plaster immobilization for 8–10 weeks, pain medication as needed, X-ray checkup at 4–8 weeks, and monitoring and adjustment of EHL FVIII doses. Bilateral hip-neck or femoral supracondylar fractures are rare, especially in individuals with haemophilia. A comprehensive approach and one-stage surgical intervention are recommended for these debilitating conditions despite the demanding and costly therapy involved. However, appropriate and timely management can help avoid the significantly higher costs associated with staged orthopaedic surgery.

**Figure 3:**
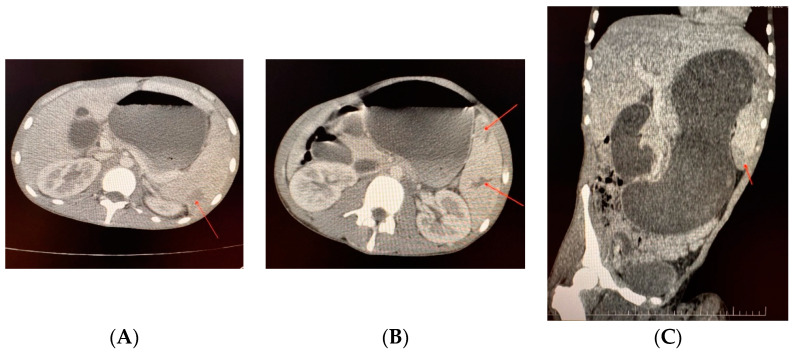
(**A**–**C**) Case 3—Abdomen and pelvis computed tomography scan with contrast (anteroposteriorly and sagittal view). Hepatosplenomegaly. Right liver lobe of 20 cm, left liver lobe at the axillary line. The kidneys and pancreas were normal. Gastric dilatation. Spleen enlarged 15/6/19 cm, with mid-body capsular fissure, two hypo-capturing areas at the inferior pole (9/2/3 cm) and posterior median region (3/3 cm), and subcapsular hematoma. Without abdominal-pelvic collections.

**Case 3.** A 19-year-old patient with severe haemophilia B, treated on-demand, was admitted to the clinic due to severe abdominal pain after trauma. The pain was located in the left hypochondrium, mesogastrium, and epigastrium. Upon clinical examination, the patient was found to have massive abdominal distention associated with a large hematoma on the left elbow and calf and debilitating arthropathy of both knees. An abdominal and pelvis computed tomography (CT) scan showed gastric dilatation, an enlarged spleen, and two splenic hematomas located at the mid-body and inferior pole. (Figure 3A–C). The patient’s haemoglobin levels dropped to 3 g/dL, and with the family’s consent (belonging to the Jehovah’s Witnesses), the patient received four units of packed red blood cells (pRBCs). The intervention was performed after draining 2.5 L of bloody stomach fluid, administering coagulation factor IX product, and ligating the splenic artery and vein: splenectomy was conducted, and a 1.5 kg large spleen was removed, revealing a small rupture in the middle of the spleen’s capsule. The patient initially recovered well after the surgery. However, on the third-day post-operation, minor bleeding was observed at the lower part of the surgical incision, along with a hematoma on the left calf and cutaneous necrosis on the left elbow hematoma. Consequently, a simultaneous surgical intervention was decided upon, which included draining the minimal supra-aponeurotic hematoma located inferior to the surgical incision and evacuating a large (0.4 L) hematoma in the left calf, along with excising the necrotic skin lesion (6/4 cm) on the left elbow and partially covering it with skin taken from the left thigh. The patient’s recovery was successful under the standard treatment regimen with FIX concentrate, and he was discharged after 27 days. The total cost for hospitalisation, medication and consumables was EUR 32,763.70 (92.80% for plasma-derived FIX concentrate). In all cases we respected the dosages for substitution as recommended in surgical interventions (Table 3); in the period of reha-bilitation, in order to prevent traumatic bleedings connected to the physical activity, we aimed at least 10–15 UI/dl of coagulation factor level.

The age of patients was different (50, 39, 19 years in case 1,2,3, respectively) without impact on substitution regimen; the amount and duration of therapy was related mostly to the severity of comorbidities and of their therapeutic intervention, factors also deciding the duration of hospital stay.

## 4. Discussion

In the field of haemophilia, the advent of impressive new therapeutic agents has opened doors for many treatment regimens based on the high levels of personalisation faced by patients, families, and caregivers. The availability, accessibility, and affordability of medicines have a decisive role, especially in the case of rare, expensive disorders like haemophilia. The problem of overlapping simultaneous and simultaneous/concurrent surgery in elective patients is a topic for decisions of national medical commissions or colleges of surgeons, being generally considered inappropriate [3,4]. It is, with controversies and debate, also the indication for simultaneous bilateral (SBOP) or multiple orthopaedic procedures to be performed by a single surgeon in a single patient with a single anaesthesia [19,20,30,31]. They are driven in clinical practice by economic reasons. Pharmacoeconomics is a valuable topic for evaluating the cost-effectiveness and affordability of therapeutical products and treatment approaches, especially in countries with limited, restrained medical resources. Therefore, it was also considered for that reason the decision of multiple simultaneous major joints—knee (SKA) or hip (SHA) arthroplasty in PwH.

There is an excellent experience with SBOP in non-hemophilic patients around the world. The first reports on patients without haemophilia with SBOP compared to staged ones emerged in 1978 [8], which, together with other reports issued between 2012 and 2013, consider this intervention safe and cost-effective [12,13,14,16,18]. However, the literature on this subject is controversial. Some other reports performed on large cohorts of patients showed inferior results, underlining higher perioperative mortality and a higher rate of neurological and cardiac complications, pulmonary embolism, and deep vein thrombosis [10,11,12,15,17]. It was also mentioned that these complications can be managed with proper perioperative care [22].

Along with this experience, considering mainly economic reasons, SBOP was also initiated in PwH. For over a decade, many cross-sectional studies and systemic meta-analyses have been undertaken on these subjects, aiming at a comparative evaluation of such a procedure with staged intervention (usually at least six months post-first arthroplasty). The main statistical differences revealed fewer costs (replacement products, duration of hospitalization, duration of sick leave), shorter hospital stay, and earlier return home and resumption of daily living activities. Additionally, a more efficient rehabilitation process has been specified that avoids the onset of therapy-induced inhibitors and lower single anaesthesia risks. Most authors reported a lack of significant differences in early and late outcomes and complications [19,20,22,23,24,25,26,32,33]. As such, they concluded that SBOP is a good, cost-effective, and not unreasonable option even in PwH.

However, in this group of patients, similar controversies have occurred regarding SBOP. Some important voices expressed concerns about the safety of this procedure, considering the bilateral elective TKA/THA to be unadvised and raising some false expectations [30,31]. The cohort of patients having undergone such an approach is more limited in Europe and reported more in Asia/South America. The data are still scant, and the number of PwH experiencing such intervention is too low for a robust statistical evaluation. Therefore, there are still contradictory opinions regarding the indication of SBOP for programmed PwH. Despite significantly higher costs, it is considered the best solution to adopt a prudent attitude and avoid this tedious approach in elective patients [19,20,30,31]. Further large-scale studies are required to obtain a definitive conclusion. Although SBOP appears safe and cost-effective in the short and medium term, the potential risks must be considered before the intervention is decided upon. If accepted, this type of surgery has to be performed in selected referral centres with significant surgical experience and optimal cooperation with expert haematologists and rehabilitation teams [19,20].

In emergency-driven situations, the decision is not a subject of debate; the concomitant simultaneous multi-joint or organ intervention is mandatory. Such interventions are frequently reported in non-haemophilic patients. These modern advanced approaches are addressed mainly to the situations of concomitant diagnosis of a severe cardiac disease with a neoplastic disease, both requiring a timely solution. There are reports of mitral valve replacement and esophageal cancer excision, surgery for breast, stomach, tongue, and colon cancer, and coronary artery bypass graft or thoracoscopic lung wedge resection and coronary bypass [27,28,29]. Despite the doubts, uncertainties, and controversies, such interventions are also accepted for haemophilia. In these situations, the immediate and remote impact on life and quality of life can be dramatic. These patients should be the subjects of long-term, complex, and rigorous follow-ups to promptly solve the prominently reported complications.

## Figures and Tables

**Table 1:** Major surgical interventions performed between June 2001 and June 2024.

Type of Intervention	One-Stage	Simultaneous	Total
**Orthopedic**	**188**	**2**	**190**
**Non-orthopedic**			
• Stomatological	29		29
• Abdominal	7		7
• Urological	6		6
• Neurosurgery	4		4
• Gynecology	2		2
• Thoracic	3		3
• Maxillofacial	6		6
• Cardiac–mitral valve replacement	1		1
• Skin graft and hematoma excision	0	1	1
• Others	13		13
**Total**	**259**	**3**	**262**

**Table 2:** The patient’s characteristics during simultaneous surgical emergency procedures.

Characteristics	Comorbidities	Emergency	Intervention	Blood Loss	Total Estimated Costs	Complications	Outcome
**Case 1.** 50-years-old male, with severe haemophilia B	-known and treated for epilepsy -chronic viral hepatitis C	-trauma-related bilateral femoral neck fracture	-bilateral simultaneous hip arthroplasty -the right hip joint is complicated with pertrochanteric fracture, needing an additional osteosynthesis with screws	~1200 mL (3 units pRBCs)	~86,856.05 € (91.75% for recombinant FIX concentrate)	No	Good, discharged after 30 days
**Case 2.** 39-years-old male, with severe haemophilia A	-chronic viral hepatitis C	-post-traumatic bilateral femoral supracondylar fracture	-open reduction and osteosynthesis with plates and screws of the pelvic limb bilaterally	~1300 mL (4 units pRBCs)	~40,217.44 € (71.78% for EHL FVIII concentrate)	No	Good, discharge after 21 days
**Case 3.** 19-years-old male with severe haemophilia B	-chronic viral hepatitis C	-intense pain in the left hypochondrium, epigastrium, and mesogastrium -massive abdominal distension -splenomegaly with two hematomas in the mid-body and inferior pole, and suspected rupture in two times -left elbow and calf hematoma with cutaneous necrosis	-splenectomy -evacuation of the left elbow hematoma -elbow skin necrosis excision (defect 6/4 cm) followed by partial covering, with skin collected from the anterolateral and 1/3 middle side of the left thigh -evacuation of the left calf hematoma, approximately 400 mL sero-hematic liquid + clots (fibrillar rupture of internal gemini muscles)	~1500 mL (4 units pRBCs)	~32,763.70 € (92.80% for plasma-derived FIX concentrate)	-acute functional gastric dilatation due to pyloric spasm, with evacuation of 2.5 L of gastric stasis liquid -hematoma at the inferior pole of the surgical wound.	Good, discharge after 27 days

pRBCs—packed red blood cells, EHL—extended half-life.

**Table 3:** The plasma level of FVIII/IX required in major surgical interventions.

	Factor VIII/IX Plasma Level Required (%)	Duration of Treatment (Days)
Pre- and intra-operatively	80–100%	
Postoperatively	60–80%	1–7
	30–50%	7–14
	15–30%	Rehabilitation period

## Data Availability

Details regarding the study’s results, as well as links to publicly archived datasets utilized or generated during the research, are not available. The unavailability of the data is attributed to privacy or ethical constraints.
